# Probing Intraband Carrier Dynamics in Degenerately N‑Doped PbS Quantum Dots and Their Implications in Optoelectronic Devices

**DOI:** 10.1021/acs.jpclett.6c00623

**Published:** 2026-04-17

**Authors:** Rajesh Bera, Mariia Shevchenko, Mariona Dalmases, Gaurav Kumar, Gerasimos Konstantatos

**Affiliations:** † Institut de Ciències Fotòniques (ICFO), 172281 Barcelona Institute of Science and Technology, 08860 Castelldefels, Barcelona, Spain; ‡ Institució Catalana de Recerca i Estudis Avançats (ICREA), 08010 Barcelona, Spain

## Abstract

Tunable intraband transitions in colloidal quantum dots (QDs) have emerged as a promising platform for electronic transitions relevant to mid- to long-wavelength infrared optoelectronic devices. However, their broad device applications have been limited by an incomplete understanding of their spectroscopic and electrical properties. In this study, we investigate both the optical and electrical characteristics of N-doped PbS QDs using ultrafast mid-infrared transient absorption spectroscopy and temperature-dependent carrier transit time measurements. Our findings reveal intraband absorption coefficients ranging from 6 × 10^3^ to 13 × 10^3^ cm^–1^, accompanied by varying carrier recombination pathways associated with different doping levels. Notably, the average carrier relaxation lifetime is 88 ps for 5.4 nm QDs, whereas highly doped 7.7 nm QDs exhibit a significantly shorter carrier lifetime of 20 ps. These results not only elucidate the intraband optical properties of PbS QDs but also provide valuable insights for comparing carrier lifetimes to carrier transit times, thereby offering guidance for the design of future mid-infrared optoelectronic devices.

## Graphical abstract

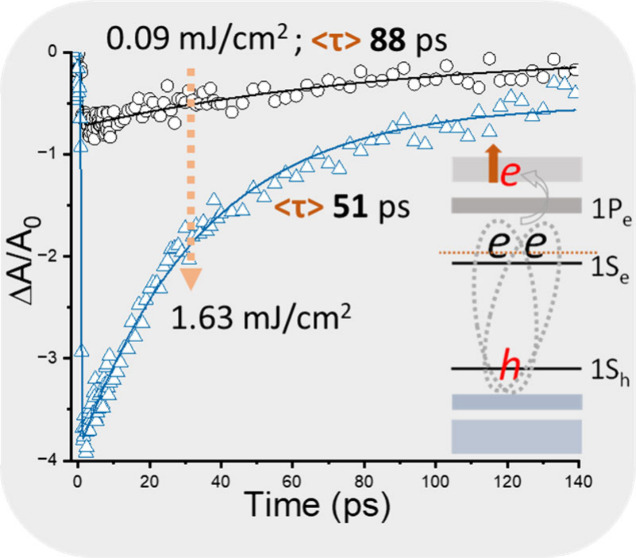

Colloidal quantum dots (QDs) have recently revolutionized solution-processed optoelectronics owing to their size-tunable electronic structure, high oscillator strengths, and comparable charge transport properties.^1–6^ Among these materials, PbS QDs have been extensively investigated for decades, particularly with respect to their interband transitions between the valence and conduction bands.^7–9^ Such transitions have enabled a broad range of applications, including high-efficiency solar cells, near-infrared (NIR) light-emitting diodes, and short-wave infrared (SWIR) photodetectors.^10–12^ However, as optoelectronic technologies shift toward the mid-infrared (MIR) and long-wave infrared (LWIR) spectral regions, there is an increasing need for QDs that exhibit stable narrow gap electronic transitions in this regime. Nevertheless, difficulties in synthesis limit the extension of bandgap transitions to such low energies, further restricting the application of QDs in infrared optoelectronic devices that rely on interband transitions almost exclusively to Hg chalcogenide QDs.

The discovery of steady-state intraband transitions, which occur between discrete electronic states within the same band, have emerged as a promising route to access the MIR and LWIR regimes.^13,14^ In contrast to interband transitions, which are constrained by the bulk bandgap of PbS (0.41 eV), intraband transitions in N-doped PbS QDs enable optical absorption at significantly lower energies (∼0.10–0.22 eV). This capability is particularly attractive for applications in thermal imaging, molecular sensing, and environmental monitoring, where conventional epitaxial infrared technologies remain prohibitively expensive. Despite the substantial technological promise of intraband transitions, the performance of PbX-based CQD intraband photodetectors^15^ remains elusive compared to other CQD counterparts in the same wavelength range.^16–18^ Although recent reports have further characterized steady-state intraband absorption and doping levels,^19^ the ultrafast behavior of these excitations particularly under high carrier density conditions and their characteristic lifetimes remains unexplored. In order to understand the origin and explore the limits of N-doped PbS CQDs, a fundamental understanding of the underlying physics of intraband states (1S_e_–1P_e_), particularly their carrier dynamics, is needed.

In this work, we address this knowledge gap by performing femtosecond two-color pump–probe spectroscopy on N-doped PbS QDs. By selectively exciting the valence band and probing the corresponding mid-infrared intraband resonances, we provide the first detailed investigation of intraband carrier dynamics. In addition to that, we performed a temperature-dependent field effect transistor (FET) measurement to evaluate the mobility and transit time of carriers in the QD film.

We synthesized PbS colloidal quantum dots (CQDs) of different sizes following the hot-injection method.^20^ The detailed synthesis procedure is provided in the Supporting Information. Transmission electron microscopy (TEM) images show average diameters of 5.4 ± 0.49, 5.9 ± 0.51, 6.8 ± 0.84, 7.4 ± 0.72, 7.7 ± 0.79, and 8.3 ± 0.66 nm (Figure S1). The N-type doping of the as-synthesized CQDs was performed following a previously reported procedure with minor modifications (Figure S2 of the Supporting Information).^15,19^ Figure 1A shows the absorbance spectra of N-doped PbS CQDs, where a clear shift in the peak position is observed with the particle size. The broad absorption band spanning 2000–1000 cm^–1^ (5–10 μm) corresponds to intraband transitions in the conduction band after N doping, which may depend on size.^19^ To check the size dependency of the intraband energy (Δ*E* = *E*_1P_e__ – *E*_1S_e__) and find out the origin of the low energy transition, we have plotted Δ*E* as a function of the size and compared it to the theoretical values extracted from the *k*·*p* model based on the quantum mechanical consideration (eq 1), where *E*_g_, α_1_, *E*_p_, and *R* denotes the bulk bandgap (=0.41 eV), the first zero of the first-order Bessel function (=4.493), the kane parameter (=13.7 eV) and the radius of QDs, respectively.^21–25^ The obtained data show that the theoretical values are in good agreement with the experimental results (Figure 1B).

1 $$E_{\mathrm{intra}}=\sqrt{\frac{{E_{g}}^{2}}{4}+\frac{2E_{p}\hbar^{2}{\alpha_{1}}^{2}}{6m_{0}R^{2}}}-\sqrt{\frac{{E_{g}}^{2}}{4}+\frac{2E_{p}\hbar^{2}\pi^{2}}{6m_{0}R^{2}}}$$

Here, three sizes of N-doped QDs (5.4 ± 0.49, 6.8 ± 0.84, and 7.7 ± 0.79 nm) were chosen because their distinct intraband peak positions lie within the measurement range of our mid-infrared transient absorption spectrometer for an ultrafast kinetic study. The average number of electrons occupying the conduction band was calculated following our previously reported method (Figure S3).^26^ The calculations indicate that the 5.4 ± 0.49, 6.8 ± 0.84, and 7.7 ± 0.79 nm QDs contain approximately 2, 5.7, and ∼8 electrons per dot, respectively, after doping. This clearly suggests that the lowest energy state of the conduction band (1S_e_) can hold a maximum of 8 electrons after N doping due to the 8-fold degeneracy originating from the nature of the band structure.^23^ In addition, we observe the high degree of bleaching in 1S_h_–1S_e_, 1S_h_–1P_e_, and 1P_h_–1P_e_ transitions in 7.7 ± 0.79 nm QDs compared to other 5.4 ± 0.49 and 6.8 ± 0.84 nm QDs after N doping (Figure 1C–E and Figure S4), suggestive of partial occupation of the 1P_e_ state at room temperature due to heavy doping in large-size QDs. Consequently, the 7.7 ± 0.79 nm QDs may exhibit a combination of 1S_e_ → 1P_e_, 1S_e_ → 1D_e_, and 1P_e_ → 1D_e_ transitions after N doping. In principle, the 1S_e_ → 1D_e_ transition is forbidden because it violates parity and the angular-momentum selection rule (Δ*l* = ±1), but the asymmetric shape of colloidal QDs may permit weak 1S_e_ → 1D_e_ transitions, although the oscillator strength is expected to be significantly lower than that of the allowed transitions.^27^ Therefore, distinguishing among the three possible steady-state transitions in the 7.7 ± 0.79 nm QDs is challenging, especially given the small energy difference between the 1S–1P and 1P–1D transitions (Figure 2A).^28^ Thus, we assign 1S_e_ → 1P_e_ and 1P_e_ → 1D_e_ for the intraband transition in highly doped QDs, since the 1S_e_ → 1D_e_ transition has a much lower transition probability (Figure 2A). Furthermore, we performed infrared variable angle spectroscopic ellipsometry (IR-VASE) to get the complex refractive index (*n̂* = *n* + *ik*) of the three N-doped QD samples, where “*n*” is the real part of the refractive index and “*k*” denotes the extinction coefficient. We have collected the data at three different positions of the films and then fitted them all using multiple models to extract the average constant values in each case (Figure 2B and C). A larger *k* value of 0.82 was recorded for the fully doped 7.7 ± 0.79 nm QDs compared to those of other lower doped samples (*k* = 0.35–0.6). Furthermore, it is noted that the *k* (0.4–0.8) value of the intraband is much higher for doped PbS than the value of undoped QDs in the interband region, reported elsewhere (Figure S6).^29^ Zhang et al. reported the *k* value of 0.6 in the intraband region (mid-IR) for the doped HgTe QDs, which is close to our results.^17^ The resulting intraband absorption coefficient (4π*k*/λ) of doped 5.4, 6.8, and 7.7 nm QDs is in the range of 6 × 10^3^, 11 × 10^3^, and 13 × 10^3^ cm^–1^, respectively, offering a highly absorbing MWIR material platform.

**Figure 1:**
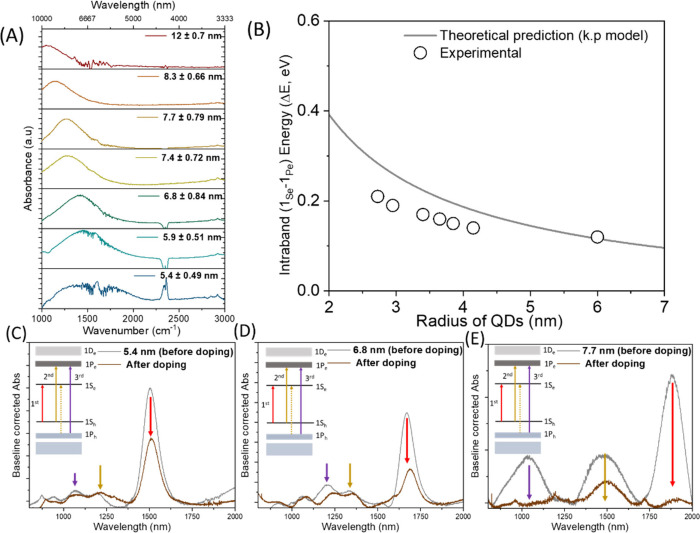
(A) Intraband absorbance spectra of PbS QD films after doping and (B) four-band *k*·*p* model of intraband energy (Δ*E*) as a function of the size and comparison to experimental results. (C–E) Baseline-corrected absorbance spectra of three different sized PbS QDs before and after doping. In the inset, the possible electronic transitions in QDs. Unprocessed data have been given in Figure S4 of the Supporting Information.

**Figure 2:**
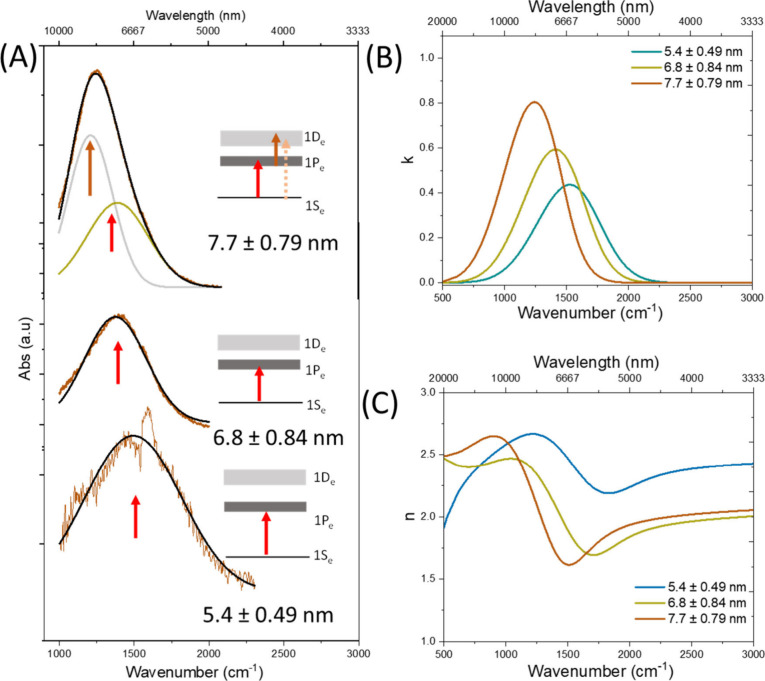
(A) Gaussian-fitted intraband transitions of three different sized QDs and possible transitions where the energy levels (1S_e_, 1P_e_, and 1D_e_) are manifold degenerate states. (B and C) Optical constant (*n* and *k*) values of three PbS QDs after doping, extracted from ellipsometry data.

To analyze the elemental composition and chemical states in PbS QDs before and after N doping, we performed X-ray photoelectron spectroscopy (XPS) on both undoped and doped samples. Figure 3A–C shows the characteristic Pb 4f peaks separated by 4.85 eV due to spin–orbit coupling. The deconvoluted peaks at 138.11 eV (4f_7/2_) and 142.96 eV (4f_5/2_) correspond to Pb–S and Pb–I bonds.^30^ The absence of additional features near the tails of these peaks indicates that no significant amount of Pb–O or Pb–OH remains after ligand exchange. Similar results were obtained for all three sizes of the QDs examined. Upon N doping, a pronounced shift in the Pb 4f binding energies is observed for every QD size. Both the 4f_7/2_ and 4f_5/2_ peaks shift to lower energies (136.86 and 141.74 eV, respectively), accompanied by the appearance of broad, low-intensity satellite peaks near 137.94 and 142.73 eV (Figure 3A–C, bottom). This shift to a lower binding energy strongly supports N doping, which modifies the local chemical environment of Pb atoms. The S 2p and I 3d peaks also shift to a lower binding energy after doping (Figures S7 and S8). In addition, the appearance of the Al 3d peak at 74 eV confirms the presence of Al in doped samples (Figure S9).^14^ The broad spectral features near the Pb 4f region originate from Pb–O–Al interactions formed during Al_2_O_3_ deposition. Therefore, careful analysis of the peak of O 1s is necessary. Deconvolution of the O 1s region reveals three components at 530.71, 531.9, and 532.71 eV, assigned to the O–Pb, O–Al, and trace OH–Pb bonds, respectively.

**Figure 3:**
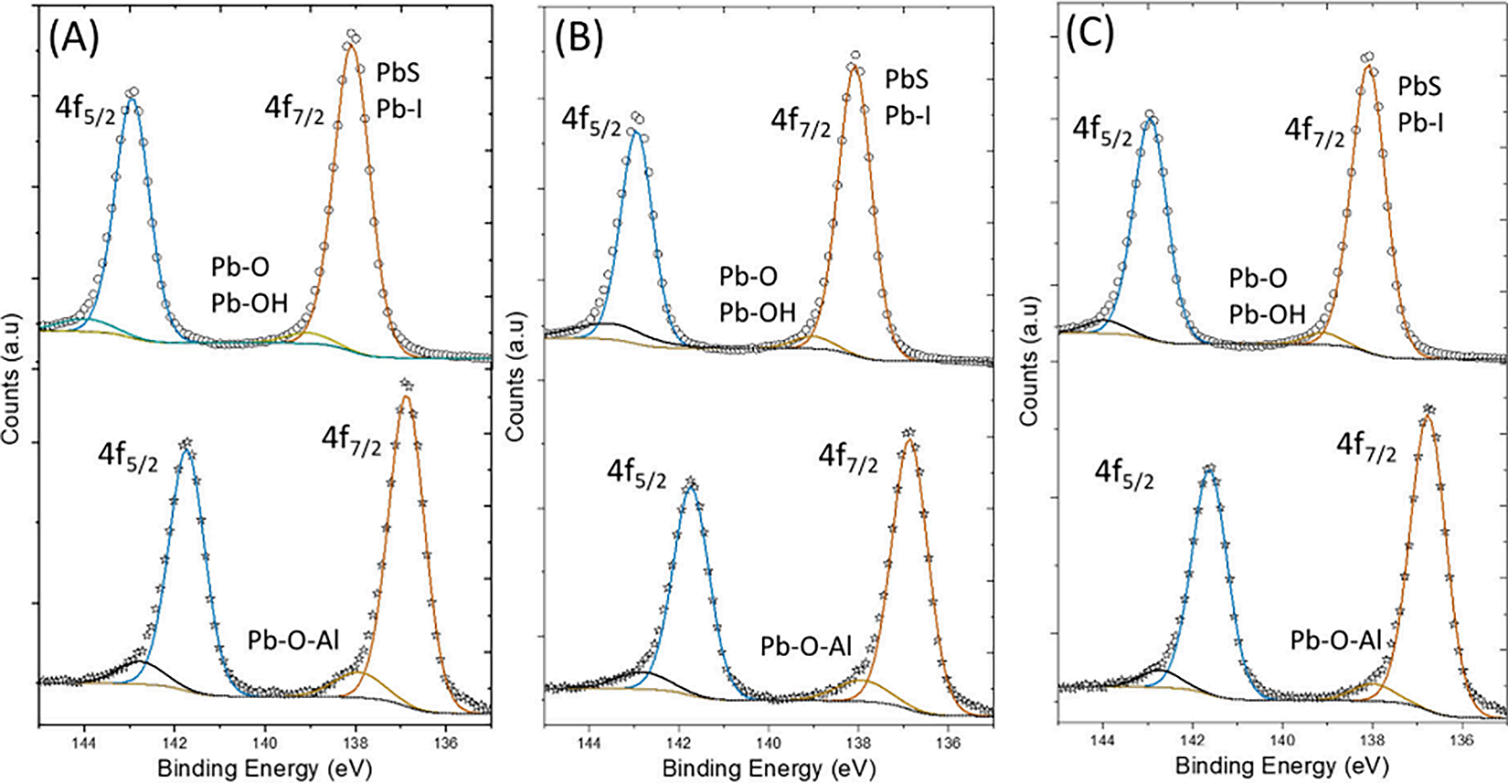
Pb 4f XPS spectra of three sizes (A) 5.4 nm, (B) 6.8 nm, and (C) 7.7 nm QDs before doping (top) and after N doping (bottom). All XPS spectra are corrected with respect to the standard C 1s peak.

To investigate the excited-state dynamics of intraband excitons in N-doped PbS QDs, we performed femtosecond two color pump–probe spectroscopy. A 1030 nm pump pulse was used to excite the samples, while the probe wavelength was tuned to the corresponding intraband absorption peaks in the mid-infrared region. As the pump photon energy at 1030 nm (∼1.2 eV) is much larger than the intraband transition energy (∼0.12–0.20 eV), direct excitation of the 1S_e_ → 1P_e_ intraband transition by the pump pulse is not possible with our experimental setup. Instead, the pump pulse excites valence band electrons into higher energy states of the conduction band. We measured the transient intraband absorbance change Δ*A*(λ,*t*) (Δ*A* = *A*_*t*_ – *A*_0_), as a function of the pump–probe delay time (*t*) and wavelength (λ), where *A*_0_ is the absorbance in the absence of the pump and *A*_*t*_ is the absorbance at a given probe delay after photoexcitation. To probe possible multicarrier interactions, we performed fluence-dependent kinetic studies on three different QD sizes, for which the number of doped electrons in the conduction band varies from 2 to ∼8 and the carrier density ranges from 1.75 × 10^19^ to 2.5 × 10^19^ cm^–3^. The kinetic traces were normalized at long delay times to clearly visualize the ultrafast processes occurring immediately after excitation (Figure 4A–C). Upon closer inspection of the early time dynamics, the decay traces of each fluence do not show significant changes throughout the experiment (Figure S10). In general, we have previously observed drastic changes in early time interband (bandgap) kinetics in conventional visible and infrared QDs when monitoring fluence-dependent dynamics.^31^ In this study, we observe an immediate bleach of the intraband (1S_e_–1P_e_) transition for the 5.4 nm QDs. In contrast, the 6.8 and 7.7 nm QDs exhibit a delay before reaching the maximum intraband bleach (Figure S10). To extract the time constants (τ) from the decay profiles, we fitted the data by using multi-exponential functions for each QD size. For the 5.4 nm QDs, a single-exponential decay with a time constant of ∼88 ps is observed at low pump fluence (Table S2). Interestingly, at higher fluences, two decay components emerge, and their values remain nearly constant across the fluence range. The faster component (31–38 ps) dominates the dynamics with a large amplitude (80–88%), while the slower component (150 ps) contributes a much smaller amplitude (12–20%). In the case of the 6.8 nm QDs, the extracted time constants differ significantly. At low fluence, only a single decay component of ∼35 ps is observed. At higher fluences, a rise component (3–7 ps) appears, followed by a decay component that decreases from ∼34 to ∼26 ps with increasing fluence. Notably, the 7.7 nm QDs exhibit a rise component (τ_rise_) even at low fluence (0.11 mJ/cm^2^). Additionally, the decay time constants are shorter compared to the smaller QDs and decrease from ∼20 to ∼13 ps with increasing fluence. To elucidate the possible relaxation pathways in the three different QD sizes, we discuss the following scenarios.

**Figure 4:**
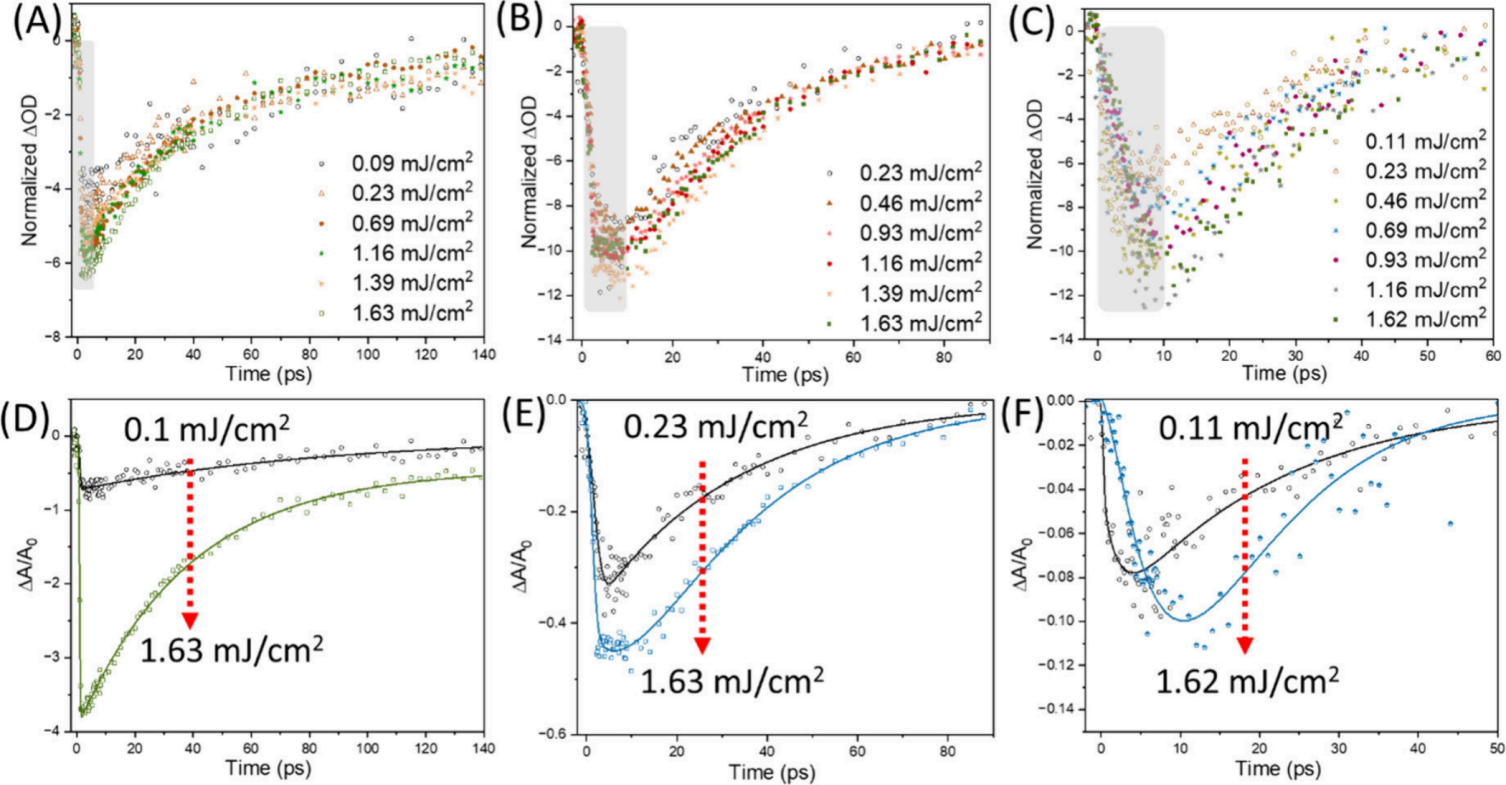
Normalized decay at a long time scale of three different sized N-doped PbS (A) 5.4 nm ± 0.49 nm, (B) 6.8 nm ± 0.84 nm, and (C) 7.7 nm ± 0.79 nm QDs upon excitation at 1030 nm at different fluences and probing at 6300, 6950, and 7900 nm, respectively. (D–F) Normalized fitted decay kinetics with Δ*A* divided by *A*_0_ at lower and higher excitation.

When QDs are doped with an electron in the conduction band and the pump pulse generates a single exciton, the photogenerated hole can be rapidly annihilated by the pre-doped 1S_e_ electrons (Scheme 1). This process releases excess energy, which can be either transferred to the photoexcited electron in a higher energy state or emitted as a photon corresponding to the bandgap energy. However, bandgap emission from fully N-doped QDs upon visible light excitation has not been observed at room temperature.^13,32^ Therefore, the more likely scenario is that the excess energy is transferred to another excited electron in a higher energy state. This hot electron subsequently relaxes back to the 1S_e_ state after thermalization via electron–phonon coupling, followed by the 1P_e_ → 1S_e_ transition. Previously, Guyot-Sionnest and co-workers assumed the hole transfer time to be effectively instantaneous (τ_hole_ ≈ 0) when explaining time-resolved intraband photoluminescence excited by a 1053 nm pump pulse.^21^ In addition, the annihilation of the photogenerated hole in the valence band can also occur via trap electrons, a process that is extremely fast and effectively creates an empty state (hole) in the 1S_e_ level. Kaifeng and co-workers reported a trap-assisted Auger process in N-doped CdSe QDs.^33^ We believe that the annihilation process in doped PbS QDs occurs on a time scale close to the instrumental resolution (∼110 fs), which explains the instantaneous formation of the intraband bleach observed for the 5.4 nm QDs upon excitation with a higher energy pump pulse (1.2 eV). It is worth noting that higher pump fluence generates multiple excitons in the 5.4 nm QDs, which further increases the number of empty states in the conduction band through the same relaxation pathways described above. Consequently, an increase in the intraband bleach amplitude is observed with increasing fluence up to a certain limit (Figure 4A).

**Scheme 1:**
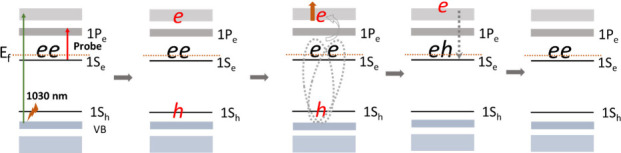
Possible Mechanism with Excited QD Films and the Consequent Bleach of the Intraband Observed

We now turn to larger QDs (6.8 and 7.7 nm), for which the number of doped electrons in the conduction band is higher compared to that in the 5.4 nm QDs. In this case, we consider that the conduction band initially contains more than two electrons, and a single exciton is generated by the pump pulse. The annihilation of the valence band hole is extremely fast and further accelerated by the presence of multiple electrons in the conduction band. As a result, empty states (holes) can be created in the conduction band through the relaxation pathways described above (Scheme 1).

Interestingly, in highly doped QDs, we observe a slow buildup of the intraband bleach at an early time (Figure S10). The decay profile resembles the kinetics of HgTe QDs while probing at the bandgap (2 μm) upon exciting above the band edge.^34^ One might expect a prompt bleach due to the ultrafast multicarrier interaction. Moreover, the high carrier density in larger QDs is expected to enhance electron–electron scattering and Auger processes.^35,36^ To understand this behavior, we examine the multi-exponential fits of the decay profiles for each fluence (Figure S11). In all cases, we primarily observe one rise component (2–7 ps) followed by one decay component. As we probe only intraband peaks, the appearance of a rise component may be due to the thermalization of hot electrons into the 1P_e_ state. These hot electrons can be generated either directly by the pump pulse or as a consequence of fast Auger processes (Scheme 1). The subsequent cooling of hot electrons via electron–phonon interactions may delay the population buildup in the 1P_e_ state, resulting in the observed slow rise of the intraband bleach. This cooling process may be further obstructed by the presence of pre-existing cooled electrons occupying the 1P_e_ state. Another possibility is that the strong coupling between adjacent QDs in films enhances the density of states near the intraband edge, which can promote the delocalization of hot electrons. Consequently, this may lead to slower cooling of hot electrons into lower lying 1P_e_ states.^34^ We observe faster carrier relaxation in larger QDs compared to smaller QDs, which is consistent with the idea that, in strongly coupled larger QDs, the wave functions of energy states overlap more efficiently with neighboring QDs, thereby increasing the number of available relaxation pathways.

Furthermore, upon examination of the normalized bleach amplitude (Δ*A*/*A*_0_) of the three samples, clear differences are observed in the *y*-axis amplitudes. The smallest QDs exhibit the largest negative bleach, whereas the bleach amplitude decreases with an increasing QD size (Figure 4D and E). It is worth noting that the intraband extinction coefficient (*k*) is higher for fully doped 7.7 nm QDs, and one might therefore expect a stronger intraband bleach amplitude. To note, the intraband bleach occurs here by an indirect process that depends on the efficiency of annihilation of pump-generated holes in the valence band. As the samples are excited with a 1030 nm pump pulse and the fluence is gradually increased, a substantial number of photogenerated carriers are created in higher energy states of both the conduction and valence bands. This leads to faster annihilation processes due to the increased number of possible recombination pathways between pre-doped electrons (1S_e_) and photogenerated holes (VB). However, the 1P_e_ state of 7.7 nm QDs is likely already occupied by doped electrons at room temperature, which results in rapid refilling of the empty 1S_e_ state by 1P_e_ electrons. This rapid refilling suppresses the population depletion of the 1S_e_ state, leading to a significantly reduced bleach of the intraband transition. Consequently, we observe a much lower nonlinear bleach amplitude (Δ*A*) in the highly doped QDs. Another possibility of having the reduced bleach amplitude may be the overlap of excited-state absorption features with the bleach signal, which is difficult to distinguish spectrally.

We know that not only is the carrier lifetime (τ_lifetime_) of QDs an important parameter, but the carrier transit time (τ_transit time_) in QD films is also crucial for fabricating efficient photodetector devices, with the ratio of τ_lifetime_/τ_transit time_ determining the responsivity of the detector. Therefore, to estimate the transit time in QD films, we performed field effect transistor (FET) measurements of N-doped PbS QD films (Figure 5A, details are provided in the Supporting Information). The mobility of each sample was first determined at different temperatures (Figures S12 and S13). It is noteworthy that 5.4 nm QDs show a much steeper change in mobility with an increasing temperature, reaching a value of 0.04 cm^2^ V^–1^ s^–1^ at 300 K from 0.007 cm^2^ V^–1^ s^–1^ at 80 K. On the other hand, highly doped 7.7 nm QDs show mobility of 0.02 cm^2^ V^–1^ s^–1^ at 80 K that reaches 0.044 cm^2^ V^–1^ s^–1^ at 300 K. A hopping-like transport mechanism is expected in these N-doped samples due to energetic disorder between adjacent QDs. The corresponding transit time are calculated following eq 2, where μ, *L* (10 μm), and *V* (1 V) denote the mobility, channel length, and applied voltage, respectively. We observe that the transit time increases with a decreasing temperature in each set of QDs (Figure 5B). Interestingly, the transit time (22–23 μs) is nearly identical at 300 K for all size, but at 80 K, it increases to 125, 80, and 43 μs for 5.4, 6.8, and 7.7 nm QDs, respectively. In general, the transit time increases at a low temperature due to reduced carrier mobility arising from thermally activated hopping and trap-limited transport in QD films. Accordingly, the activation energy (*E*_a_) may determine how strongly trap-limited transport depends on the temperature. However, a very low activation energy indicates degenerate doping in the conduction band with no evidence of trap-assisted transport for the intraband carriers (Figure 5C). Hence, considering the transit time in the microsecond scale and the average lifetime of intraband excitons in the picosecond scale, it would be hard to efficiently extract photoexcited carriers within this short time scale in devices.

2 $$\tau_{\mathrm{transit time}}=L^{2}/\left(\mu \cdot V\right)$$

In conclusion, we investigated the relaxation mechanisms of intraband excitons in N-doped PbS QDs of three different sizes, where the degree of doping varies systematically with the QD size. Lightly doped QDs exhibit a slower recombination dynamic, with a characteristic time constant of approximately 88 ps, whereas heavily doped QDs show a much faster bleach recovery. Notably, no evidence of trap-assisted Auger recombination was observed in the heavily doped QDs.^33^ If trap-localized electrons were responsible for annihilating valence band holes, then we would expect a prolonged lifetime of hot electrons in higher excited states, resulting in slower intraband relaxation.

**Figure 5:**
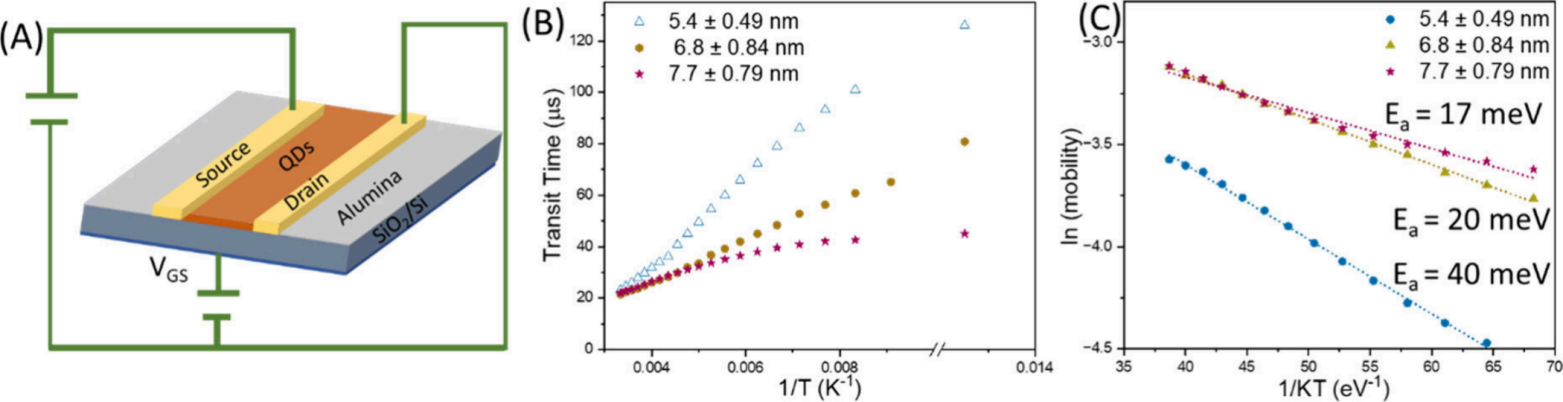
(A) Schematic of the bottom gate field effect transistor (FET). (B) Transit time of QDs as a function of the temperature. (C) Plot of mobility as a function of 1/*KT* to extract activation energy (*E*_a_) for three different sizes as mentioned in the figures.

Furthermore, the carrier transit time, which is one of the key parameters for a QD-based photodetector device, is higher at a low temperature. Although the strong absorption coefficients of the intraband transitions highlight the potential of these materials for infrared optoelectronic applications, the very short carrier lifetime in conjunction with long transit times currently limits device performance. Therefore, further improvements in prolonging the intraband carrier lifetime and reaching a higher carrier mobility are required.

## Supplementary Material



## Notes

Detailed experimental procedures, instrumentation, TEM images of CQDs, absorption spectra of undoped and doped samples, optical constants of doped and undoped samples, XPS data, normalized decay traces at an early time scale, fitted decay profiles, fitted parameters, FET source–drain current, and mobility of CQDs as a function of the temperature (PDF)
