# Supplementary material for: Probing Intraband Carrier Dynamics in Degenerately N‑Doped PbS Quantum Dots and Their Implications in Optoelectronic Devices
Source: J Phys Chem Lett. 2026 Apr 17;17(17):4974–81. doi: 10.1021/acs.jpclett.6c00623 (PMC13137249; doi:10.1021/acs.jpclett.6c00623)
Supplement: Supplementary file 1 [file jz6c00623_si_001.pdf]

**Supporting Information**

# Probing Intraband Carrier Dynamics in Degenerately N-doped PbS Quantum Dots and their Implications in Optoelectronic Devices

Rajesh Bera[‡], Mariia Shevchenko[‡], Mariona Dalmases[‡], Gaurav Kumar[‡], Gerasimos Konstantatos*[‡†]

[‡]ICFO, Institut de Ciències Fotòniques, The Barcelona Institute of Science and Technology, Castelldefels, Barcelona, 08860 Spain
[†]ICREA, Institució Catalana de Recerca i Estudis Avançats, Barcelona, 08010 Spain.

*Email: gerasimos.konstantatos@icfo.eu

**Experimental Section**

**Chemicals**: Lead (II) oxide (PbO, 99.999%) was purchased from Strem Chemicals. 1-octadecene (ODE, 90%) was acquired from Thermo Scientific Chemicals. Oleic acid (OA, 90%), hexamethyldisilathiane (HMS, synthetic grade), 1-Ethyl-3-methylimidazolium iodide 97% (EMII) and butylamine (BA, 99.5%) were sourced from Sigma Aldrich, and acetone, ethanol, and anhydrous toluene were obtained from Scharlab. All chemicals were used as received.

**Lead sulfide colloidal quantum dots (PbS CQDs) synthesis**: PbS CQDs were synthesized using the hot-injection method under an inert atmosphere. A mixture of PbO, ODE, and OA was heated at 100°C under vacuum for 1 hour to form lead oleate. Subsequently, the atmosphere was switched to argon, and the temperature was set to the desired reaction temperature. Several solutions of HMS in ODE were then injected, either rapidly or dropwise, depending on the specific synthesis. After the final HMS/ODE solution was injected, the crude solution was allowed to cool naturally to room temperature. For purification, the PbS QDs were precipitated by adding a mixture of acetone and ethanol and were subsequently re-dispersed in anhydrous toluene. If the solid did not re-disperse properly after precipitation, a small volume of BA was added to aid stability. This purification process was repeated two more times. Finally, the solution's concentration was adjusted to 20 mg/mL, and it was stored in a glovebox under an inert atmosphere. The specific conditions for each PbS CQD synthesis are summarized in Table S1. The synthetic method used for quantum dots with excitonic peaks at 1950 nm and 2200 nm was based on a previously published procedure.[1]

**N- doping of CQDs**:

The CQD solution (20 mg/mL in toluene) was spin coated onto $CaF_2$ substrates at 2000 rpm. The native oleic acid ligand was then exchanged with EMII ligand (7 mg/mL in methanol), followed by rinsing with dry methanol at 2500 rpm to remove excess organic residues. This process was repeated three times to obtain the desired film thickness. All layer-by-layer (LBL) deposition steps were carried out inside a glovebox to prevent oxidation. Subsequently, an $Al_2O_3$ layer was deposited at 60 °C by atomic layer deposition (ALD)[2] to ensure stable N-doping of PbS and to protect the films from aerial oxidation.

**FET measurements:**

Devices were fabricated using a single channel gold electrode (800 µm × 10 µm) patterned on alumina ($Al_2O_3$) coated $SiO_2$ (300 nm)/Si substrates (Figure 5A). A 56 nm thick $Al_2O_3$ layer was deposited on the top of $SiO_2$ to suppress any current leakage to the doped Si bottom gate. The films were deposited by spin coating the QDs samples three times to achieve the desired

thickness. The dielectric constants of alumina and $SiO_2$ are taken as 8 and 3.9, respectively.[3] Electrical measurements were carried out by sweeping the gate voltage ($V_{GS}$) at different temperatures while applying a constant drain-source voltage ($V_{SD}$) of 1V. We observed clear modulation of the source-drain current when sweeping the gate voltage in the transistor based on 5.4 nm QDs (Figure S12). In contrast, the transistors utilizing 6.8 nm and 7.7 nm QDs exhibited quasi linear transfer characteristics with negligible response to the gate. This loss of gate modulation is attributed to the high doping density in the larger QDs, which leads to electrostatic screening of the gate field. Carrier mobility was extracted from the transfer characteristics curve ($I_D$ vs $V_{GS}$) following the equation (1-2) where $I_D$, $\mu$, $C_i$, $V_{GS}$, $V_{SD}$, $\epsilon_r$, $t_{dielectric}$, W, L, denotes the drain current, mobility, capacitance per unit area of the gate dielectric, gate voltage, constant drain voltage applied during the scan, relative permittivity of oxides, thickness, width and length of channel respectively. Figure S9 shows that the mobility of all three size QDs systematically increases with increasing temperature.

$$I_D = \frac{W}{L}\mu C_i V_{SD} V_{GS} \qquad \text{......................} \ (1)$$

$$C_i = \frac{\epsilon_0 \ \epsilon_r}{t_{dielectric}} \qquad \text{.................} \quad (2)$$

**Instrumentation**

UV-vis-NIR absorption measurements were performed with the Cary 5000 UV-vis-NIR spectrophotometer in solution phase and absorption of film samples were done in Nicolet iS50R FTIR instrument.

Transmission electron microscopy (TEM) images were acquired using a JEOL 2100 microscope operating at an accelerating voltage of 200 kV at the Scientific and Technological Centres of the University of Barcelona (CCiT-UB), Barcelona, Spain.

X-ray photoelectron spectroscopy (XPS) data were collected using a SPECS PHOIBOS 150 hemispherical analyser (SPECS GmbH, Berlin, Germany) equipped with a monochromatic Ka X-ray source (1486.74 eV) under ultra-high vacuum conditions ($10^{-10}$ mbar). The data were fitted by Voigt function with Shirley type background after calibration to the C 1s spectra at a binding energy of 284.8 eV.

Transient absorption spectroscopy (TAS) was carried out using an infrared fs-transient absorption spectrometer (Helios, Ultrafast System). Samples were excited using 110 fs pulses at 1030 nm generated by the amplified 800 nm Ti:Sapphire laser (Spectra-Physics, 1 kHz) through nonlinear frequency mixing in an optical parametric amplifier. The pump fluence was controlled by a reflective IR filter. To generate the probe wavelength, a nonlinear crystal has

been used for producing mid infrared wavelength following the non-linear difference frequency generation (DFG) method. A precise motorized stage was utilized to regulate the delay time between the pump and probe. Two probe signals, (one is for sample and another is for reference) is collected by a multipixel MCT arrays (2 × 32) detector (Infrared Associates, Inc.) operated at low temperature. Both probe signal helps to minimize the error during real time fluctuation. Surface Xplorer software has been used to fit the kinetic traces.

The electrode patterning on the $Al_2O_3$ deposited Si substrate was done using the conventional photo lithography techniques (Maskless Aligner Heidelberg MLA 150) followed by etching, developing and metal deposition (Lesker thermal evaporation) process.

Atomic layer deposition (ALD) of alumina was done using Arradiance GEMStarXT series model. High-purity trimethylaluminium (TMA), purchased from STREM Chemicals Inc., was used as aluminium (Al) precursor. Pure $H_2O$ was used as O precursor. The deposition was carried out at 60 ºC. The TMA and $H_2O$ manifolds were maintained at 150 ºC during gas supply. Each layer of alumina was formed by applying a 15 ms pulse of $H_2O$, followed by a 35-ms pulse of TMA.

For ellipsometry measurement we used IR-VASE Mark II ellipsometer which can cover the wide spectral range from 1.7 to 30 microns.

FET transfers characteristics were measured in a LakeShore cryogenic probe station using a Keysight B1500A Semiconductor Device Analyzer. The temperature was varied by liquid nitrogen.

**Table S1**: Specific conditions used for the synthesis of PbS CQDs of different sizes.

| 1st excitonic peak wavelength (nm) | $T_{reaction}$ (ºC) | PbO (mg) | OA (mL) | ODE (mL) | 1st injection: µL HMS/mL ODE | 1st injection: Inj. Time (min) | 2nd injection: µL HMS/mL ODE | 2nd injection: Inj. time (min) | 3rd injection: µL HMS/mL ODE | 3rd injection: Inj. time (min) | 4th injection: µL HMS/mL ODE | 4th injection: Inj. time (min) | 5th injection: µL HMS/mL ODE | 5th injection: Inj. time (min) | 6th injection: µL HMS/mL ODE | 6th injection: Inj. time (min) | 7th injection: µL HMS/mL ODE | 7th injection: Inj. time (min) | 8th injection: µL HMS/mL ODE | 8th injection: Inj. time (min) |
|---|---|---|---|---|---|---|---|---|---|---|---|---|---|---|---|---|---|---|---|---|
| **1475** | **100** | **446** | **3.8** | **50** | **75/3** Fast | **0** | **120/9** Dropwise 0.75mL/min | **6** | | | | | | | | | | | | |
| **1580** | **100** | **446** | **3.8** | **50** | **65/3** Fast | **0** | **105/9** Dropwise 0.75mL/min | **6** | | | | | | | | | | | | |
| **1650** | **100** | **446** | **3.8** | **50** | **65/3** Fast | **0** | **150/9** Dropwise 0.75mL/min | **6** | | | | | | | | | | | | |
| **1750** | **110** | **446** | **4.5** | **75** | **60/3** Fast | **0** | **120/9** Dropwise 0.75mL/min | **6** | | | | | | | | | | | | |
| **1850** | **120** | **516** | **4.8** | **100** | **40/3** Fast | **0** | **166/9** Dropwise 0.41mL/min | **6** | | | | | | | | | | | | |
| **1950** | **130** | **446** | **2.76** | **63** | **53/3** Fast | **0** | **20/2** Fast | **20** | **20/2** Fast | **32** | **30/2** Fast | **44** | **30/2** Fast | **56** | | | | | | |
| **2200** | **130** | **446** | **2.76** | **63** | **53/3** Fast | **0** | **20/2** Fast | **20** | **20/2** Fast | **32** | **30/2** Fast | **44** | **30/2** Fast | **56** | **40/2** Fast | **68** | **40/2** Fast | **80** | **40/2** Fast | **92** |

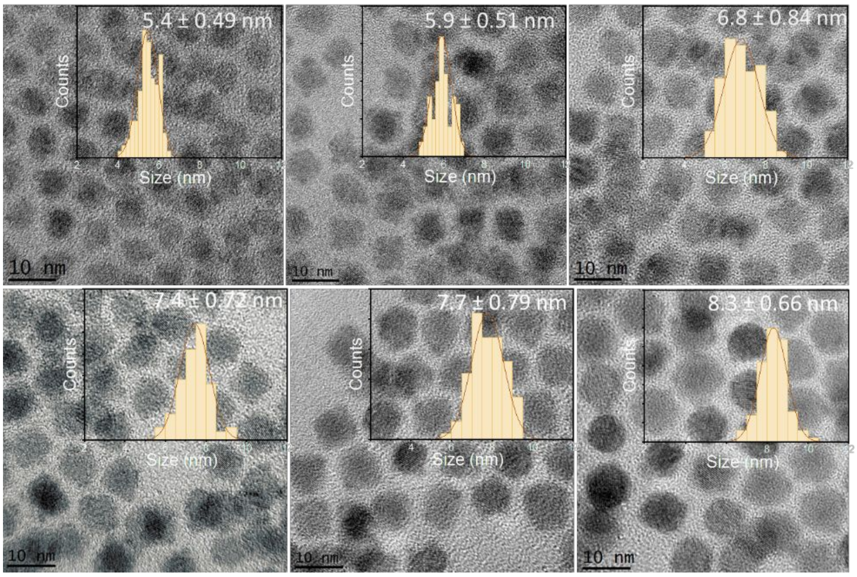


**Figure S1.** TEM images of different size PbS QDs and histogram of their corresponding size in inset.

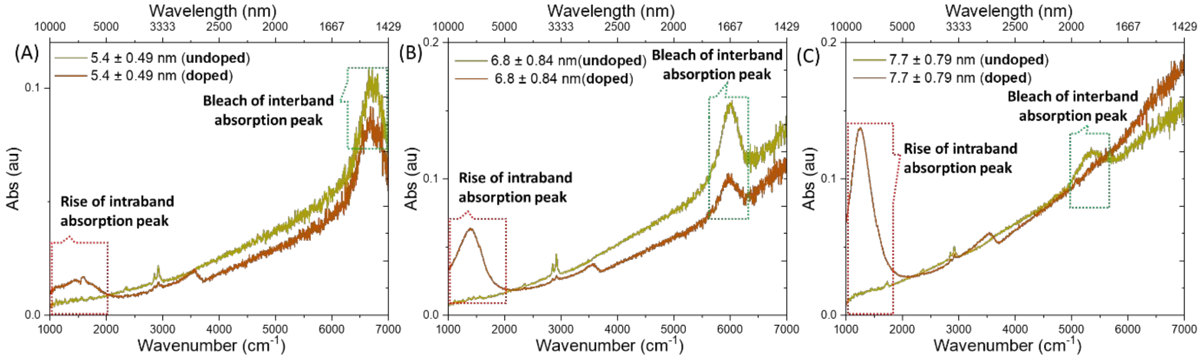


**Figure S2.** The absorption spectra of undoped and doped thin film samples (A-5.4, B-6.8 and C-7.7 nm) where we can see appearing of intraband peaks at low energy region and bleach of band gap transition in the high energy part as mentioned in each figure. The amplitude of bleach depends on the degree of doping in the conduction band. To note, the noise in the spectrum at the high energy arise as we use DTGS detector instead of InGaAs detector in our FTIR spectrophotometer to cover both band gap and intraband peaks.

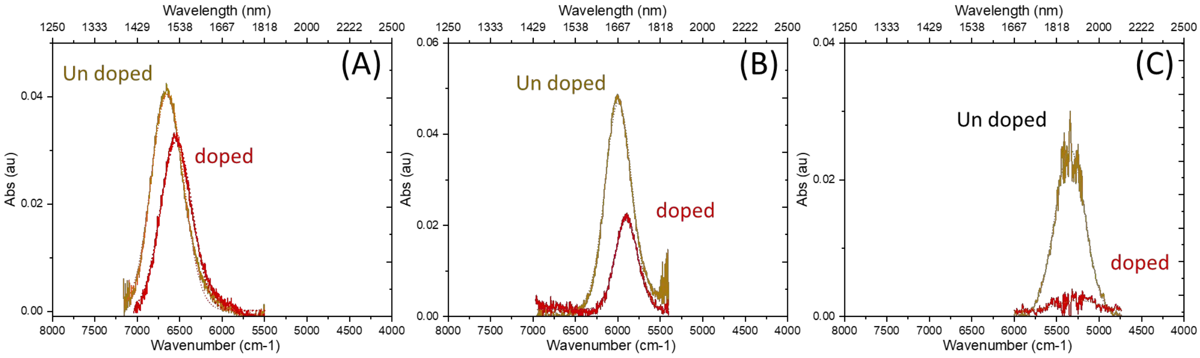


**Figure S3.** Baseline corrected band gap transition before and after doping (A- 5.4 nm, B- 6.8 nm, C-7.7 nm) to calculate the integrated area at band edge and finally number of electrons doped.

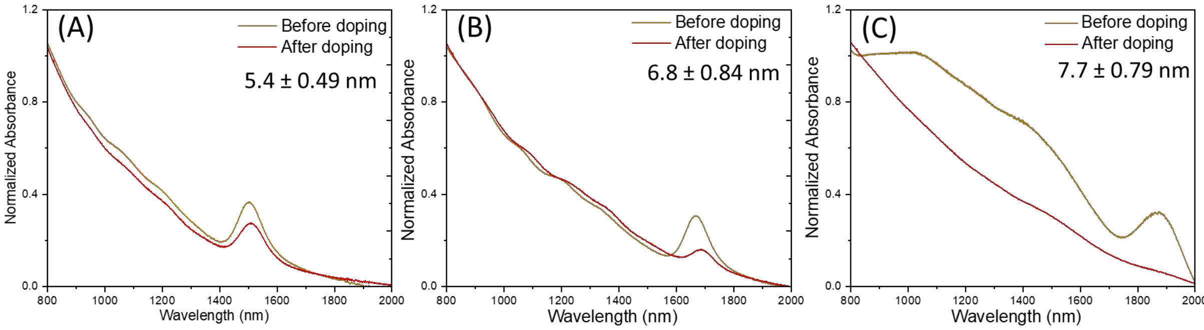


**Figure S4**. Normalized Absorbance spectra of three different size CQDs before and after doping (A-5.4 nm, B- 6.8 nm and C-7.7 nm) at higher energy where the transition would be uninterrupted by doping. Here we can see the bleach of band gap transition after doping. It is further processed to assign the corresponding transitions as mentioned in the main manuscript.

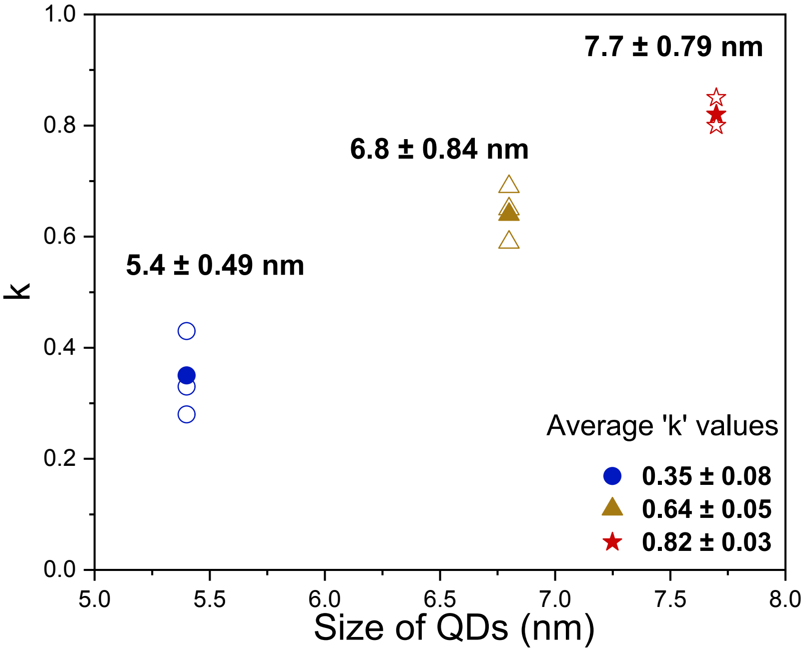


**Figure S5.** Average ´k´ values including standard deviations of three different n doped PbS QDs thin films as mentioned in the figure. Data collected at three different positions of the films and values are taken at the intraband peak maximum of the corresponding CQDs. Dark points are the average ´k´ of the corresponding samples.

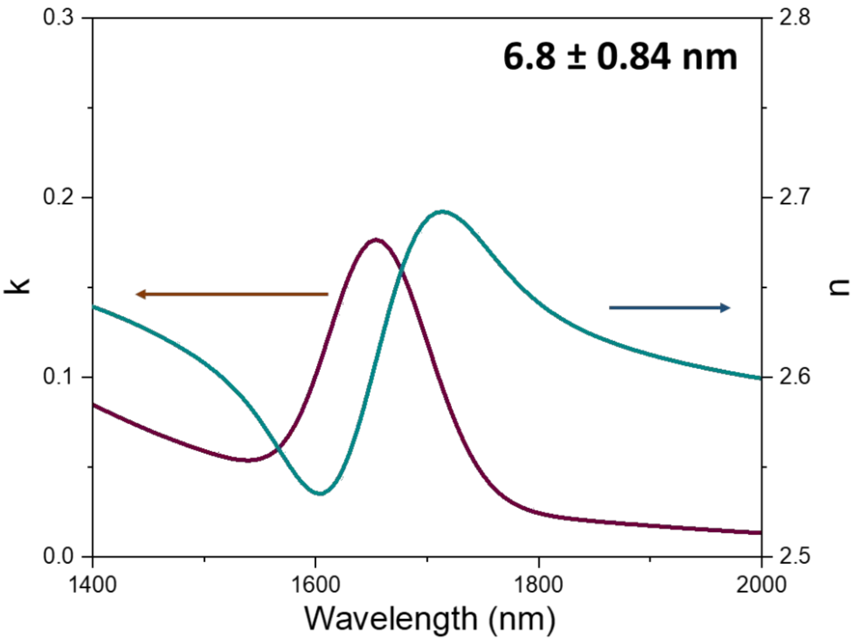


**Figure S6.** Optical constants of the undoped PbS (6.8 nm) where QD´s surface is modified by iodide (EMII) ligands.

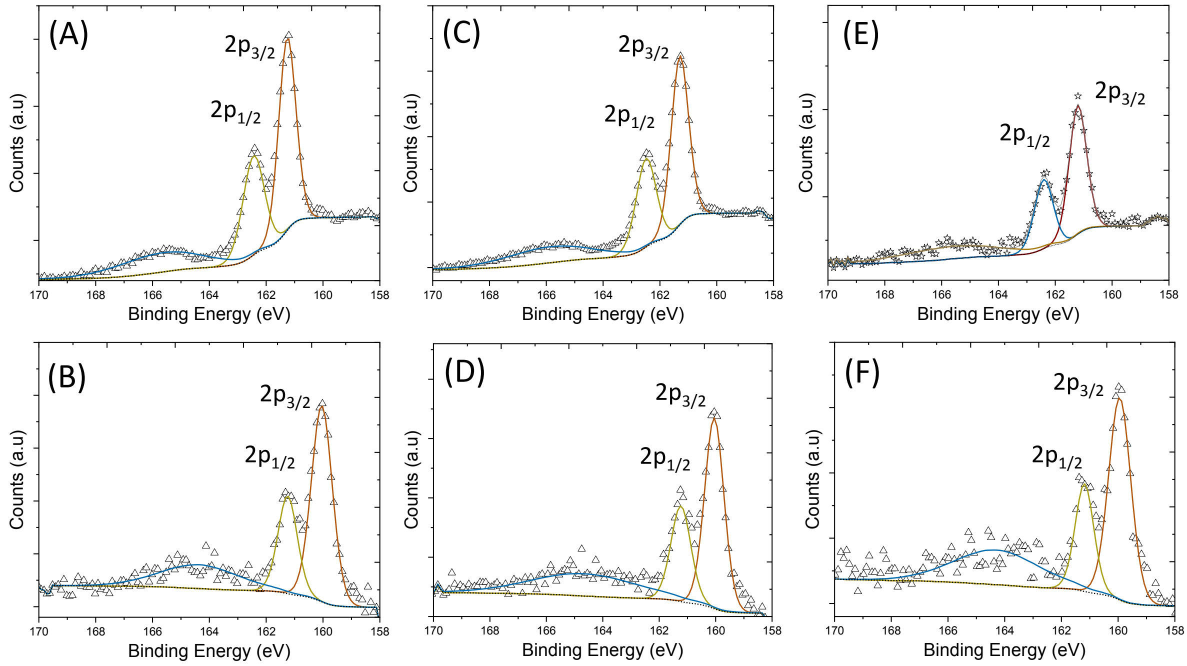


**Figure S7.** XPS spectra of S (2p) state before (up row) and after doping (down row). (A, B-5.4 nm), (C, D-6.8 nm), and (E, F- 7.7 nm).

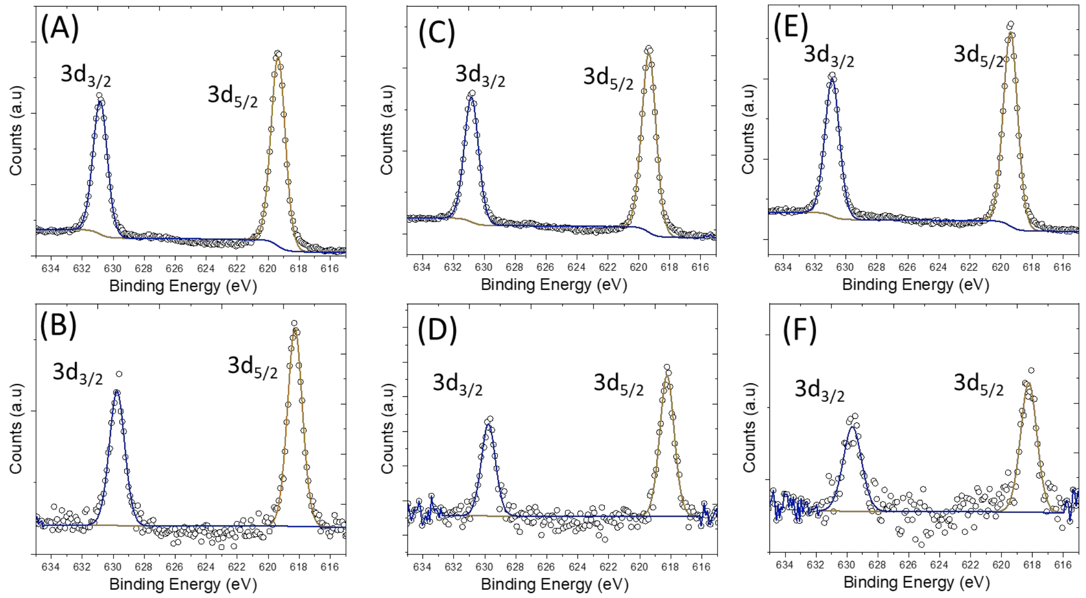


**Figure S8.** XPS spectra of I(3d) element before (up row) and after doping (down row). (A, B-5.4 nm), (C, D- 6.8 nm), and (E, F- 7.7 nm).

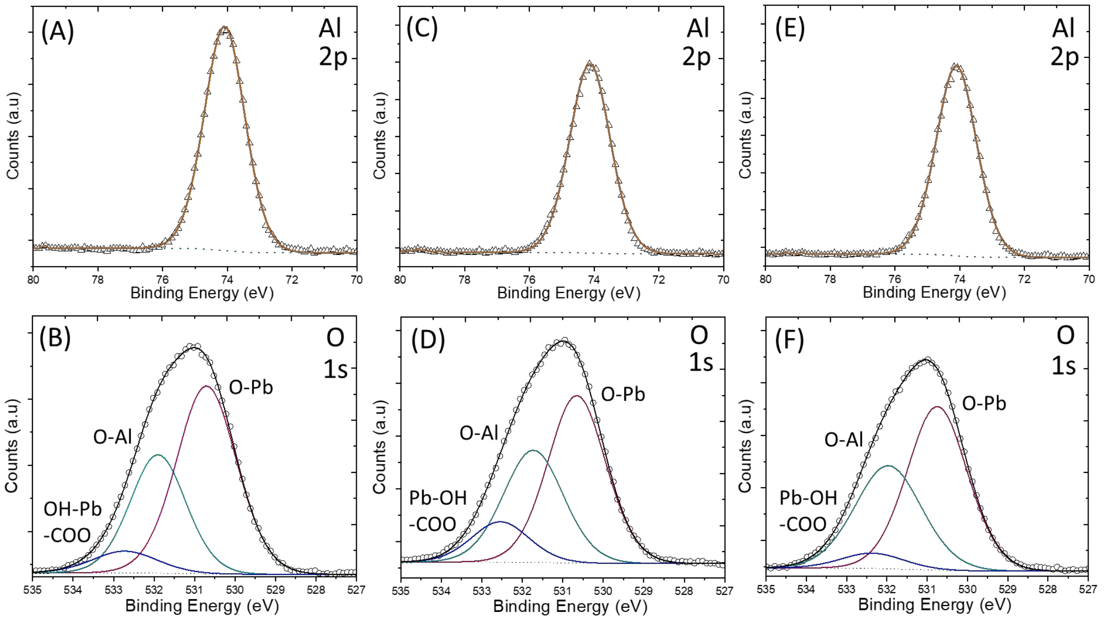


**Figure S9.** XPS spectra of Al (2p) and O (1s) atom after atomic layer deposition of $Al_2O_3$for stable N-doping. (A,B- 5.4 nm), (C,D-6.8 nm), (E,F-7.7 nm).

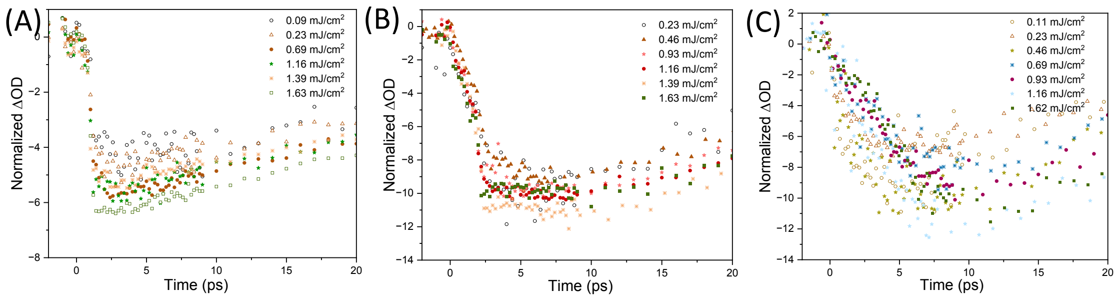


**Figure S10.** The normalized decay traces (A-5.4 nm, B-6.8 nm, C-7.7 nm) when probing at (A) 6300 nm, (B) 6950 nm and (C) 7900 nm upon exciting at 1030 nm.

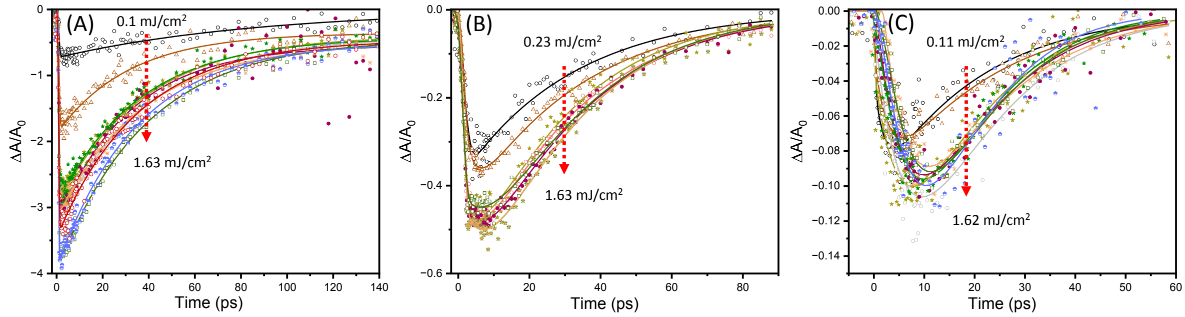


**Figure S11.** Fitted decay profile of three different size QDs (A) 5.4 nm, (B) 6.8 nm and (C) 7.7 nm when probing at 6300 nm, 6950 nm and 7900 nm respectively upon exciting at 1030 nm.

**Table S2**: Multiexponential fittings of decay profile and their corresponding parameters.

| Samples | Fluence (mJ/cm$^2$) | $\tau_{rise}$ (ps) | $\tau_1$ ($a_1$) (ps) | $\tau_2$ ($a_2$) (ps) |
|---|---|---|---|---|
| 5.4 ± 0.49 nm | 0.09 | - | 88 | - |
| | 0.23 | - | 31 (0.8) | 150 (0.2) |
| | 0.69 | - | 38 (0.87) | 150 (0.13) |
| | 1.16 | - | 36 (0.87) | 150 (0.13) |
| | 1.39 | - | 38 (0.84) | 150 (0.13) |
| | 1.63 | - | 38 (0.88) | 150 (0.12) |

| Samples | Fluence (mJ/cm$^2$) | $\tau_{rise}$ (ps) | $\tau_1$ ($a_1$) ps | $\tau_2$ ($a_2$) ps |
|---|---|---|---|---|
| 6.8 ± 0.84 nm | 0.23 | | 35 | - |
| | 0.46 | 3 | 34 | - |
| | 0.93 | 4 | 30 | - |
| | 1.16 | 6 | 29 | - |
| | 1.39 | 7 | 27 | - |
| | 1.63 | 7 | 26 | - |

| Samples | Fluence (mJ/cm$^2$) | $\tau_{rise}$ (ps) | $\tau_1$ ($a_1$) ps | $\tau_2$ ($a_2$) ps |
|---|---|---|---|---|
| 7.7 ± 0.79 nm | 0.11 | 2 | 20 | - |
| | 0.23 | 3 | 21 | - |
| | 0.46 | 5 | 18 | - |
| | 0.69 | 6 | 17 | - |
| | 0.93 | 6 | 15 | - |
| | 1.16 | 7 | 12 | - |
| | 1.62 | 7 | 13 | - |

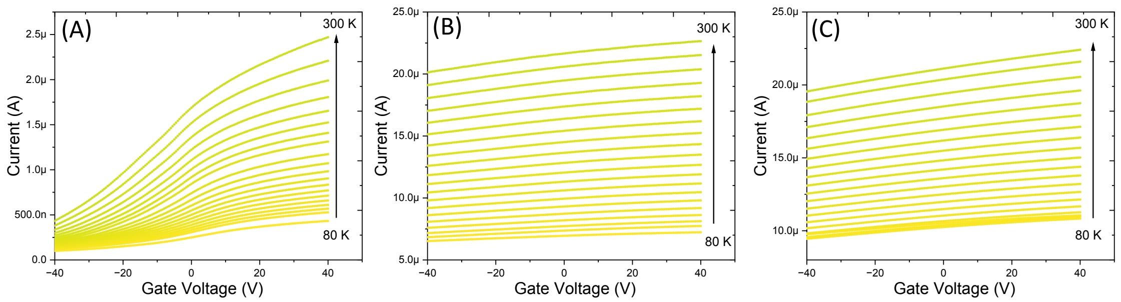


**Figure S12.** FET source-drain current under variable gate voltage and different temperature A-5.4 nm, B-6.8 nm and C-7.7 nm.

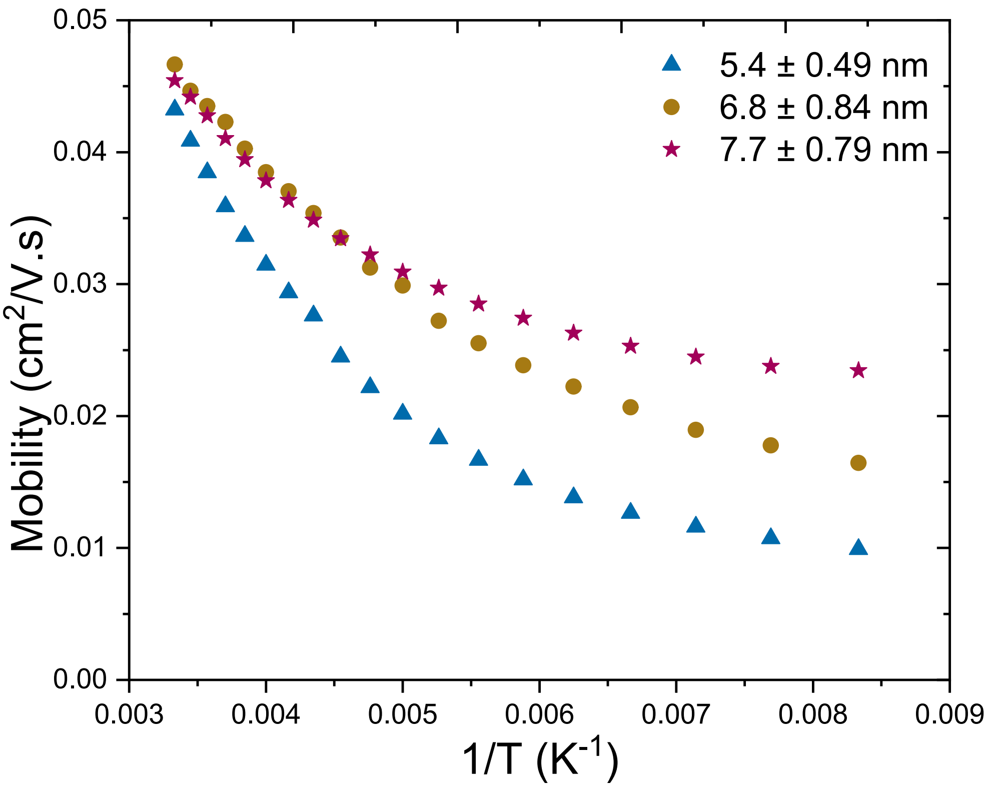


**Figure S13.** Extracted mobility of three N-doped CQDs as a function of temperature.

**References:**

(1) Dong, C.; Liu, S.; Barange, N.; Lee, J.; Pardue, T.; Yi, X.; Yin, S.; So, F., Long-Wavelength Lead Sulfide Quantum Dots Sensing up to 2600 nm for Short-Wavelength Infrared Photodetectors. *ACS Appl. Mater & Interfaces* **2019**, *11*, 44451-44457.

(2) Christodoulou, S.; Ramiro, I.; Othonos, A.; Figueroba, A.; Dalmases, M.; Özdemir, O.; Pradhan, S.; Itskos, G.; Konstantatos, G., Single-Exciton Gain and Stimulated Emission across the Infrared Telecom Band from Robust Heavily Doped Pbs Colloidal Quantum Dots. *Nano Lett.* **2020**, *20*, 5909-5915.

(3) Etinger-Geller, Y.; Zoubenko, E.; Baskin, M.; Kornblum, L.; Pokroy, B., Thickness Dependence of the Physical Properties of Atomic-Layer Deposited $Al_2O_3$. *J. Appl. Phys.* **2019**, *125*.
